# Author Correction: The T cell receptor repertoire of tumor infiltrating T cells is predictive and prognostic for cancer survival

**DOI:** 10.1038/s41467-021-24897-w

**Published:** 2021-07-22

**Authors:** Sara Valpione, Piyushkumar A. Mundra, Elena Galvani, Luca G. Campana, Paul Lorigan, Francesco De Rosa, Avinash Gupta, John Weightman, Sarah Mills, Nathalie Dhomen, Richard Marais

**Affiliations:** 1 Molecular Oncology Group, Cancer Research UK Manchester Institute, The University of Manchester, Alderley Park, Macclesfield, Cheshire, UK; 2 Medical Oncology, The Christie NHS Foundation Trust, Manchester, UK; 3 Department of Surgery, The Christie NHS Foundation Trust, previously Department of Surgical Oncological and Gastroenterological Sciences DISCOG (University of Padova), The Christie NHS Foundation Trust, Manchester, UK; 4 Immunotherapy - Cell Therapy and Biobank, Istituto Scientifico Romagnolo per lo Studio e la Cura dei Tumori (IRST) “Dino Amadori” IRCCS, Meldola, Italy; 5 Molecular Biology Core Facility, Cancer Research UK Manchester Institute, The University of Manchester, Alderley Park, Macclesfield, Cheshire, UK; 6 Manchester Cancer Research Centre Biobank, The Christie NHS Foundation Trust, Manchester, UK

**Keywords:** Cancer immunotherapy, Statistical methods, Predictive markers, Tumour immunology

Correction to: *Nature Communications* 10.1038/s41467-021-24343-x; published online 02 July 2021.

The original version of this Article contained an error in the author affiliations.

Luca G. Campana was incorrectly associated with Medical Oncology, The Christie NHS Foundation Trust, Manchester, UK.

This has now been corrected in both the PDF and HTML versions of the Article.

