# Supplementary material for: The T cell receptor repertoire of tumor infiltrating T cells is predictive and prognostic for cancer survival
Source: Nat Commun. 2021 Jul 2;12:4098. doi: 10.1038/s41467-021-24343-x (PMC8253860; doi:10.1038/s41467-021-24343-x)
Supplement: Supplementary file 1 — Supplementary Information [file 41467_2021_24343_MOESM1_ESM.pdf]

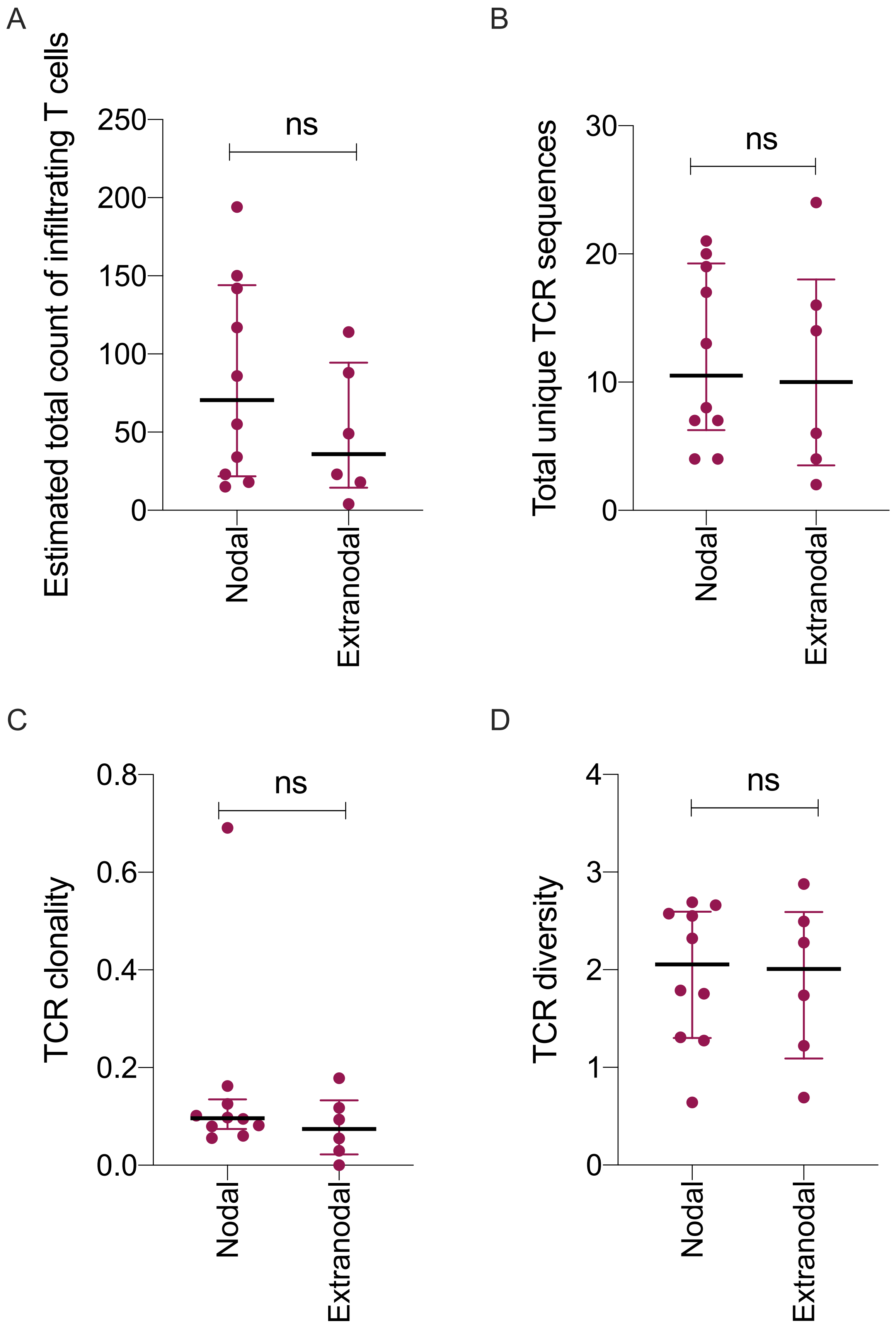

Supplementary Figure 1. TIL/Tc TCR metrics according to pre-treatment biopsy location. Comparison of TIL/Tc TCR repertoire metrics in nodal (n=10) vs extranodal (n=6)

biopsy sites. a Estimated total number of tumor TIL/Tc (median=70.5 vs 36, P=0.3), b Total unique TIL/Tc TCR sequences (median=10.5 vs 10, P=0.6156), c TIL/Tc TCR clonality (median=0.096 vs 0.074, P=0.3132) and d TIL/Tc TCR diversity (median=2.1 vs 2, P=0.7128). Mann-Whitney two-sided tests. Horizontal dotted lines represent median and standard deviation. ns is not significant, n is single patient. Source data are provided as a Source Data file.

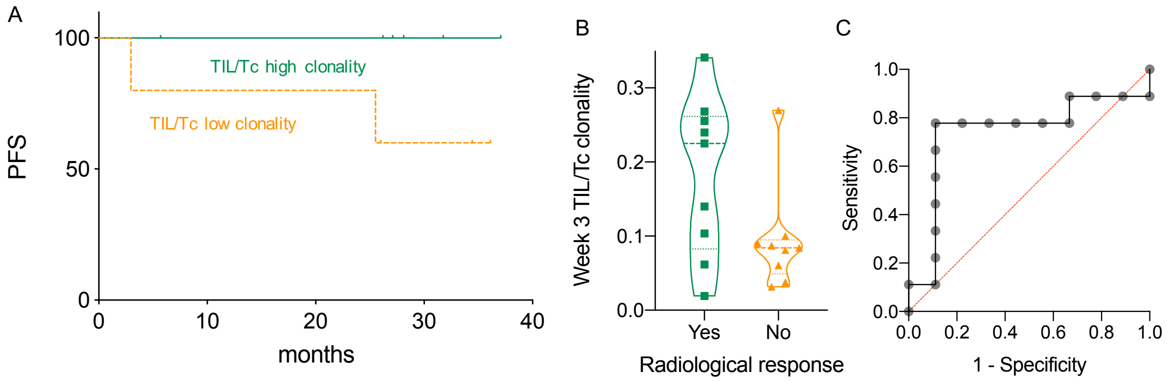

Supplementary Figure 2. TIL/Tc TCR clonality of pre-treatment and on-treatment samples of patients receiving neoadjuvant PD1 CPB. a Progression-free survival curves for an advanced melanoma cohort of patients treated with neoadjuvant PD1 CPB with high (green) or low (orange) pre-treatment TIL/Tc clonality (n=11, cut-off=median, log-rank P=0.118). b Violin plots of the on-treatment (week 3) TIL/Tc clonality distribution in patients who achieved radiological response (green, n=9, median=0.18, SD=0.11) and progressed (orange, n=9, median=0.09, SD=0.07) to treatment with neoadjuvant PD1 CPB in an advanced melanoma cohort[1] (simple logistic regression log-likelihood ratio for association with probability of response=4.2, P=0.0392, n=18). c Receiver operating curve (ROC) of the of the linear regression response prediction (area under the curve=0.74). Analyses are two-sided, TIL/Tc clonality and diversity are retained as continuous variables in the regression analyses; n is single patient; single green and orange dots represent single patients; horizontal dotted line in the violin plots represent median and SD; SD=standard deviation. Source data are provided as a Source Data file.

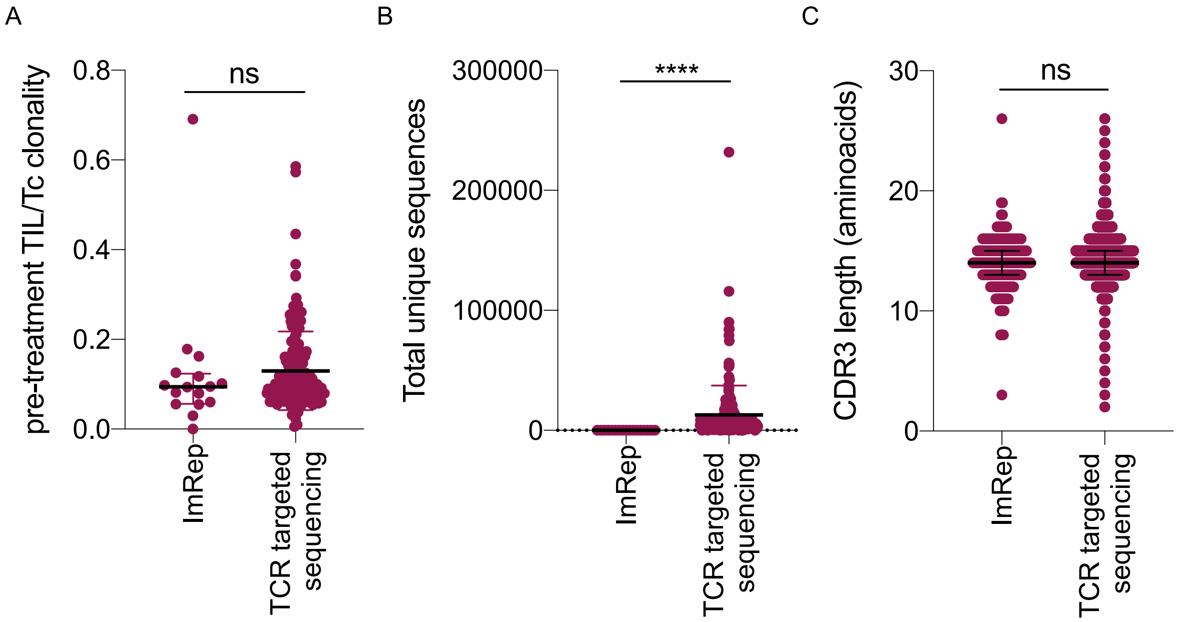

Supplementary Figure 3. Comparison of TIL/Tc TCR metrics in samples analyzed with RNA-Seq or targeted sequencing platforms. a TIL/Tc clonality in samples studied by means of ImRep analysis[2] of RNA-Seq (n=16, median=0.094, SD=0.157) and TCR targeted sequencing[1,3-5] (n=176, median=0.101, SD=0.088); Mann-Whitney test P=0.263 (ns=not significant, dot=single patient). b TIL/Tc total unique TCR sequences (clonotypes) in samples studied by means of ImRep analysis of RNA-Seq (n=16, median=10.5, SD=7.2) and TCR targeted sequencing[1,3-5] (n=176, median=5613, SD=24384); Mann-Whitney test P<0.0001 (****=P<0.0001, dot=single patient). c cumulative distribution of TIL/Tc TCR complementarity determining region 3 (CDR3) length (aminoacids) in samples studied by means of ImRep analysis of RNA-Seq (n=187, median=14, SD=2.2) and TCR targeted sequencing[1,3-5] (n=157189, median=14, SD=1.7); Kolmogorov-Smirnov test P=0.0712 (ns=not significant, dot=single TCR sequence). Analyses are two-sided, horizontal lines represent median and standard deviation. Source data are provided as a Source Data file.

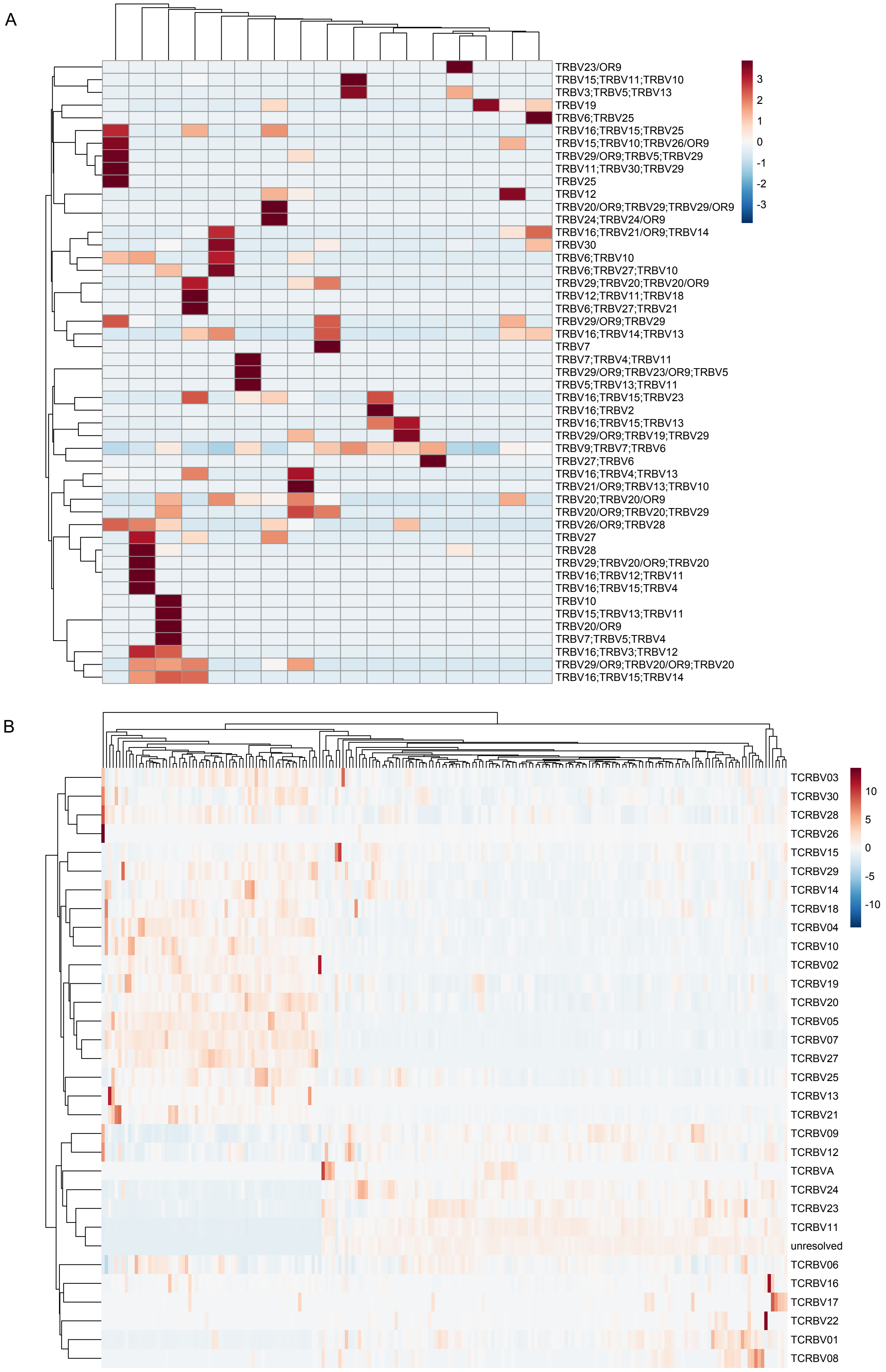
A
TRBV23/OR9
TRBV15;TRBV11;TRBV10
TRBV3;TRBV5;TRBV13
TRBV19
TRBV6;TRBV25
TRBV16;TRBV15;TRBV25
TRBV15;TRBV10;TRBV26/OR9
TRBV29/OR9;TRBV5;TRBV29
TRBV11;TRBV30;TRBV29
TRBV25
TRBV12
TRBV20/OR9;TRBV29;TRBV29/OR9
TRBV24;TRBV24/OR9
TRBV16;TRBV21/OR9;TRBV14
TRBV30
TRBV6;TRBV10
TRBV6;TRBV27;TRBV10
TRBV29;TRBV20;TRBV20/OR9
TRBV12;TRBV11;TRBV18
TRBV6;TRBV27;TRBV21
TRBV29/OR9;TRBV29
TRBV16;TRBV14;TRBV13
TRBV7
TRBV7;TRBV4;TRBV11
TRBV29/OR9;TRBV23/OR9;TRBV5
TRBV5;TRBV13;TRBV11
TRBV16;TRBV15;TRBV23
TRBV16;TRBV2
TRBV16;TRBV15;TRBV13
TRBV29/OR9;TRBV19;TRBV29
TRBV9;TRBV7;TRBV6
TRBV27;TRBV6
TRBV16;TRBV4;TRBV13
TRBV21/OR9;TRBV13;TRBV10
TRBV20;TRBV20/OR9
TRBV20/OR9;TRBV20;TRBV29
TRBV26/OR9;TRBV28
TRBV27
TRBV28
TRBV29;TRBV20/OR9;TRBV20
TRBV16;TRBV12;TRBV11
TRBV16;TRBV15;TRBV4
TRBV10
TRBV15;TRBV13;TRBV11
TRBV20/OR9
TRBV7;TRBV5;TRBV4
TRBV16;TRBV3;TRBV12
TRBV29/OR9;TRBV20/OR9;TRBV20
TRBV16;TRBV15;TRBV14
3
2
1
0
-1
-2
-3
B
TCRBV03
TCRBV30
TCRBV28
TCRBV26
TCRBV15
TCRBV29
TCRBV14
TCRBV18
TCRBV04
TCRBV10
TCRBV02
TCRBV19
TCRBV20
TCRBV05
TCRBV07
TCRBV27
TCRBV25
TCRBV13
TCRBV21
TCRBV09
TCRBV12
TCRBVA
TCRBV24
TCRBV23
TCRBV11
unresolved
TCRBV06
TCRBV16
TCRBV17
TCRBV22
TCRBV01
TCRBV08
10
5
0
-5
-10

Supplementary Figure 4. Tumor infiltrating T cells (TIL/Tc) T cell receptor (TCR) V gene usage across platforms. Non-clustered heatmaps showing the usage of TCR V family gene generated with the R package LymphoSeq in samples studied by means of (a) ImRep analysis[2] of RNA-Seq data and (b) targeted TCR sequencing with Adaptive Immunoseq.

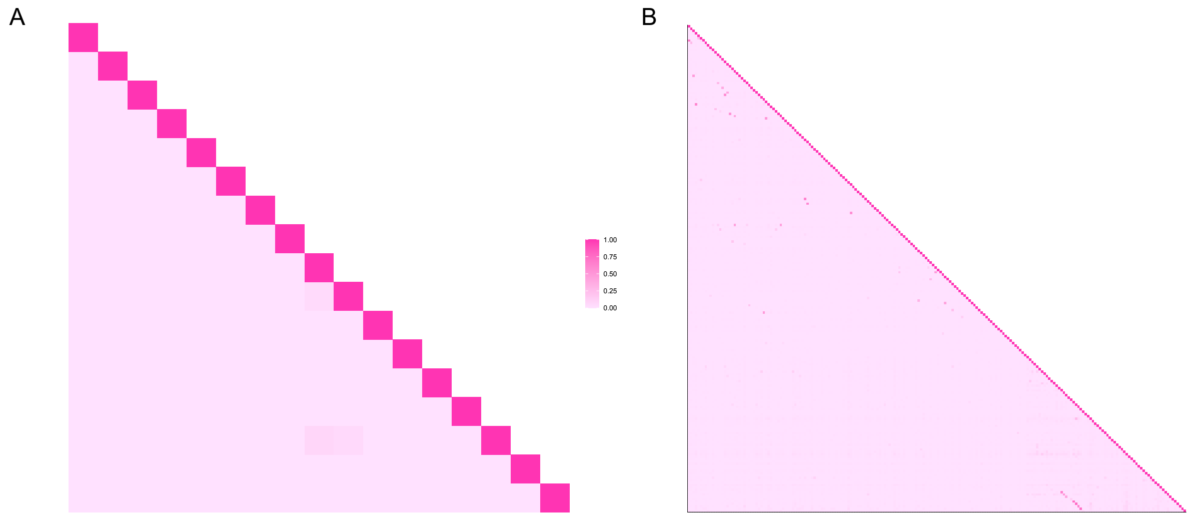

Supplementary Figure 5. Similarity matrices of the tumor infiltrating T cells (TIL/Tc) T cell receptors (TCR). Similarity matrices in samples studied by means of (a) ImRep analysis[2] of RNA-Seq data and (b) targeted TCR sequencing with Adaptive Immunoseq. The color intensity of each square represents the similarity score derived from the pairwise comparison between two samples as calculated by R package Lymphoseq, with darker shades corresponding to higher similarity scores. Source data are provided as a Source Data file.

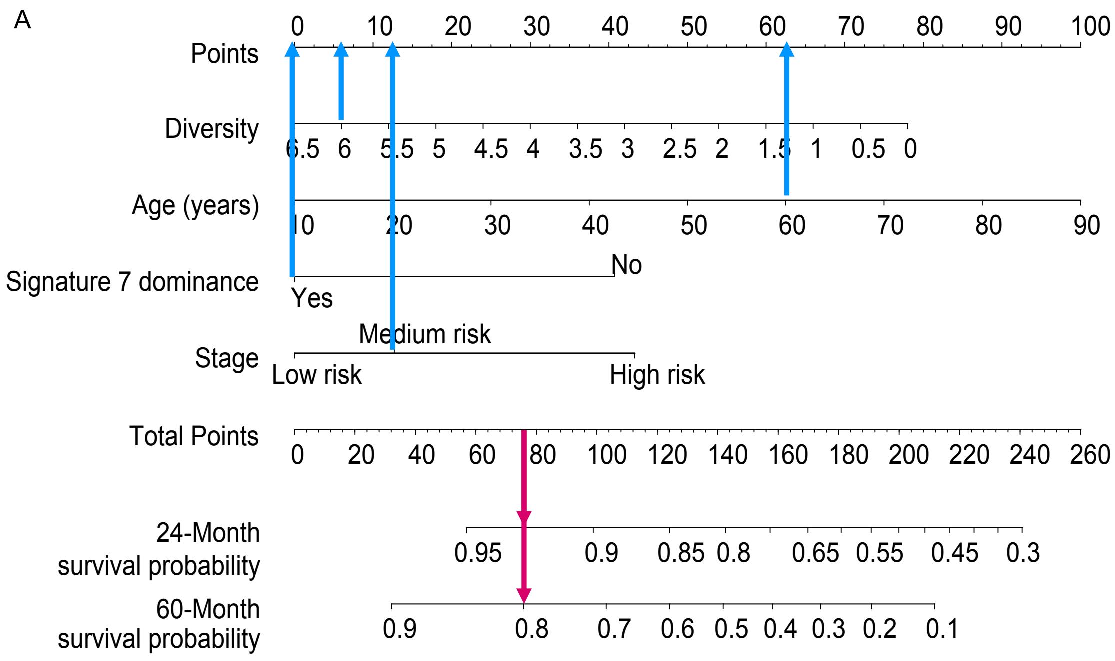

Supplementary Figure 6. Simulation of the prognostic nomogram use. a Nomogram usage for an example patient: the blue arrows indicate the hazard points corresponding to the age of a 60-year-old patient (62.5 points) diagnosed with a medium risk melanoma (12.5 points) with TIL/Tc diversity=6 (3 points) and signature 7 dominance (0 points). The points are summed and the total (78 points) is used to determine that the patient's probability (pink arrows) of being alive at 24 months is between 90 and 95% (24-month survival probability=0.90-0.95) and at 60 months is 80% (60-month survival probability=0.80).

**Supplementary Table 1. Univariate and multivariate Cox regression analysis for overall survival in melanoma patients treated with anti-PD1 drugs. Tests are two-sided.**				
**Covariates**	**Univariate Cox regression analsis**			**Multivariate Cox regression analsis**
	**HR**	**95% CI**	**P**	**P**
TIL/Tc clonality	4.84E-14	9.341E-27 0.2509	**0.0401**	**0.04867**
baseline peripheral blood LDH	1.006	1.001 - 1.012	**0.0362**	0.236
total TIL/Tc	0.9964	0.9799 - 1.013	0.682	
total TIL/Tc clones	0.96049	0.8467 - 1.09	0.531	
TIL/Tc diversity (Renyi index)	0.8516	0.3116 - 2.327	0.754	
total non-synonymous single nucleotide variations	0.999	0.9973 - 1.003	0.947	
maximum PD-L1 staining	3.27E-05	0 - inf	0.999	
Due to the small sample size the covariates were first assessed in a Cox regression univariate analysis; the covariates significantly associated with overall survival at univariate analysis (TIL/Tc clonality and baseline LDH) were analysed in a multivariate model with backward selection. Significant covariate P are highlighted in bold. LDH is				

lactic dehydrogenase; TIL/Tc is tumour infiltrating T lymphocytes; PD-L1 is programmed death ligand 1.

**Supplementary Table 2. Final Cox regressionmodel and excluded variables for SKCM TCGA cohort. Tests are two-sided.**				
**Covariates retained in the final model after deleting factors**	**Coefficient**	**Standard Error**	**Wald Z**	**P**
TIL/Tc diversity (Renyi index)	-0.2448	0.05831	-4.199	2.68E-05
Age (in years)	0.02	0.006023	3.321	8.97E-04
Low risk stage	-1.0885	0.238131	-4.571	4.85E-06
Medium risk stage	-0.8169	0.242005	-3.375	7.37E-04
High risk stage	*reference*			
Signature 7 dominance	-0.9613	0.23854	-4.03	5.58E-05
**Covariates deleted with backward selection**	**Residual**	**Degrees of freedom**	**P**	**AIC**
Tumor anatomical location	7.75	6	0.2571	-4.25
Total single nucleotide variations	7.84	7	0.3467	-6.16
TIL/Tc clonality	8.26	8	0.4088	-7.74
Total number of TCR sequences (total number of TIL/Tc clones)	8.65	9	0.4706	-9.35
Sex	9.72	10	0.4655	-10.28
Total number of TIL/Tc	11.06	11	0.4379	-10.94

Summary of the Cox regression model analysis for SKCM TCGA cohort. Disease stage is classified according to the TNM annotation in the TCGA clinical information file (low risk is stage Tis, I, IIA; medium risk is IIB, III, IIIA, IIIB and high risk is IIC, IIIC and IV). Coefficient is ln(hazard ratio), SKCM is skin cancer melanoma, TCGA is The Cancer Genome Atlas, TIL/Tc is tumour infiltrating T cells, TCR is T cell receptor.

Supplementary Table 3. Final Cox regression model and excluded variables for BRCA TCGA cohort. Tests are two-sided.

**Covariates retained in the final model after deleting factors**	**Coefficient**	**Standard Error**	**Wald Z**	**P**
Age (in years)	0.0299572	0.0076827	3.899	9.65E-05
TIL/Tc diversity (Renyi index)	-0.2445158	0.0931856	-2.624	8.69E-03
Total single nucleotide variations	0.0006206	0.0002122	2.925	3.45E-03
ER IHC positive	-0.3314766	0.2255399	-1.47	1.42E-01
**Covariates deleted with backward selection**	**Residual**	**Degrees of freedom**	**P**	**AIC**
Race category	0.44	3	0.9323	-5.56
Total number of TCR sequences (total number of TiTc clones)	0.48	4	0.9752	-7.52
TIL/Tc clonality	0.81	5	0.9767	-9.19
Ethnicity category	1.4	6	0.966	-10.6
Total number of TIL/Tc	2.47	7	0.9291	-11.53

Summary of the Cox regression model analysis for BRCA TCGA cohort. The analysis was performed including stratification for stage due to hazard hypothesys violation for stage covariate (disease stage is classified according to the TNM annotation in the TCGA clinical information file ; localised is stage I to II; advanced is III and IV). Coefficient is ln(hazard ratio); BRCA is breast carcinoma, TCGA is The Cancer Genome Atlas, ER is estrogen receptor, IHC is immune-histochemistry, TIL/Tc is tumour infiltrating T cells, TCR is T cell receptor.

Supplementary Table 4. Final Cox regression model and excluded variables for low-smoker LUSC TCGA cohort. Tests are two-sided.

**Covariates retained in the final model after deleting factors**	**Coefficient**	**Standard Error**	**Wald Z**	**P**
Age (in years)	0.04213	0.024391	1.727	8.41E-02
Karnofsky performance score	-0.007849	0.004614	-1.701	8.90E-02
TIL/Tc diversity (Renyi index)	-0.479419	0.221929	-2.16	3.08E-02
Total single nucleotide variations	-0.003339	0.001605	-2.081	3.75E-02
**Covariates deleted with backward selection**	**Residual**	**Degrees of freedom**	**P**	**AIC**
TIL/Tc clonality	0.02	1	0.9018	-1.98
Total number of TCR sequences (total number of TiTc clones)	0.26	2	0.8787	-3.74
Total number of TIL/Tc	0.4	3	0.9394	-5.6
Stage	0.94	4	0.9187	-7.06

Summary of the Cox regression model analysis for LUSC TCGA cohort of patients with cigarette use lower than the median. Disease stage is classified according to the TNM annotation in the TCGA clinical information file (localised is stage I to II; advanced is III and IV). Coefficient is ln(hazard ratio); LUSC is lung squamous carcinoma, TCGA is The Cancer Genome Atlas, TiTc is tumour infiltrating T cells, TCR is T cell receptor.

**Supplementary Table 5. Final Cox regression model and excluded variables for LUAD TCGA cohort. Tests are two-sided.**				
**Covariates retained in the final model after deleting factors**	**Coefficient**	**Standard Error**	**Wald Z**	**P**
Total number of TCR sequences (total number of TIL/Tcclones)	0.01401	0.008101	1.729	0.0837359
TIL/Tc clonality	-2.41196	1.358555	-1.775	0.0758344
TIL/Tc diversity (Renyi index)	-0.72667	0.272283	-2.669	0.0076122
Stage (localised)	-0.77692	0.201937	-3.847	0.0001194
Stage (advanced)	*reference*			
Total number of TiTc	-0.01409	0.00816	-1.727	0.084232
**Covariates deleted with backward selection**	**Residual**	**Degrees of freedom**	**P**	**AIC**
Cigarette smoking hiistory (pack/year value)	0.08	1	0.7753	-1.92
Total single nucleotide variations	0.16	2	0.9247	-3.84
Summary of the Cox regression model analysis for LUAD TCGA cohort. Disease stage is classified according to the TNM annotation in the TCGA clinical information file (localised is stage I to II; advanced is III and IV). Coefficient is ln(hazard ratio); LUSC is lung squamous carcinoma, TCGA is The Cancer Genome Atlas, TIL/Tc is tumour infiltrating T cells, TCR is T cell receptor.				
Karnofsky performance score could not be included in the analysis because missing for 386 cases.				

Supplementary Table 6. Final Cox regression model and excluded variables for TGCT TCGA cohort. Tests are two-sided.

**Covariates retained in the final model after deleting factors**	**Coefficient**	**Standard Error**	**Wald Z**	**P**
TIL/Tc diversity (Renyi index)	-0.2619	0.148	-1.77	0.07678
**Covariates deleted with backward selection**	**Residual**	**Degrees of freedom**	**P**	**AIC**
Onotree code	2.51	2	0.2845	-1.49
Total single nucleotide variations	2.93	3	0.4022	-3.07

Summary of the Cox regression model analysis for TGCT TCGA cohort. Due to the smll number of events the analysis was performed to assess the prognostic value of tumour infiltrating T cell diversity vs the the prognostic relevant tumour subset and the mutational burden. Coefficient is ln(hazard ratio); TGCT is testicular germinal cell tumours, TCGA is The Cancer Genome Atlas.

Supplementary Table 7. Final Cox regression model and excluded variables for KIRC TCGA cohort. Tests are two-sided.

**Covariates retained in the final model after deleting factors**	**Coefficient**	**Standard Error**	**Wald Z**	**P**
Age (in years)	0.0599024	0.0132011	4.538	5.69E-06
Frameshift mutations mutations	-0.0655014	0.0200127	-3.273	1.06E-03
TIL/Tc diversity (Renyi index)	-1.164806	0.5677074	-2.052	4.02E-02
Total number of TCR sequences (total number of TiTc clones)	0.0139874	0.0071725	1.95	5.12E-02
Total single nucleotide variations	0.0055219	0.0028339	1.949	5.14E-02
TIL/Tc clonality	-4.0532303	2.3526993	-1.723	8.49E-02
Total number of TIL/Tc	-0.0001762	0.0001061	-1.661	9.67E-02
**Covariates deleted with backward selection**	**Residual**	**Degrees of freedom**	**P**	**AIC**
Sex	0.08	1	0.7753	-1.92

Summary of the Cox regression model analysis for KIRC TCGA cohort, the analysis was performed with stratificationfor stage. Disease stage is classified according to the TNM annotation in the TCGA clinical information file (localised is stage I to II; advanced is III and IV). Coefficient is ln(hazard ratio); KIRC is kidney renal clear cell carcinoma carcinoma, TCGA is The Cancer Genome Atlas, TIL/Tc is tumour infiltrating T cells, TCR is T cell receptor.
