# Supplementary material for: The T cell receptor repertoire of tumor infiltrating T cells is predictive and prognostic for cancer survival
Source: Nat Commun. 2021 Jul 2;12:4098. doi: 10.1038/s41467-021-24343-x (PMC8253860; doi:10.1038/s41467-021-24343-x)
Supplement: Supplementary file 3 — Reporting Summary [file 41467_2021_24343_MOESM3_ESM.pdf]

nature research

Corresponding author(s): Richard Marais

Last updated by author(s): Apr 6, 2021

# Reporting Summary

Nature Research wishes to improve the reproducibility of the work that we publish. This form provides structure for consistency and transparency in reporting. For further information on Nature Research policies, see our Editorial Policies and the Editorial Policy Checklist.

## Statistics

For all statistical analyses, confirm that the following items are present in the figure legend, table legend, main text, or Methods section.

| n/a | Confirmed | |
|---|---|---|
| ☐ | ☒ | The exact sample size (*n*) for each experimental group/condition, given as a discrete number and unit of measurement |
| ☐ | ☒ | A statement on whether measurements were taken from distinct samples or whether the same sample was measured repeatedly |
| ☐ | ☒ | The statistical test(s) used AND whether they are one- or two-sided<br>*Only common tests should be described solely by name; describe more complex techniques in the Methods section.* |
| ☐ | ☒ | A description of all covariates tested |
| ☐ | ☒ | A description of any assumptions or corrections, such as tests of normality and adjustment for multiple comparisons |
| ☐ | ☒ | A full description of the statistical parameters including central tendency (e.g. means) or other basic estimates (e.g. regression coefficient) AND variation (e.g. standard deviation) or associated estimates of uncertainty (e.g. confidence intervals) |
| ☐ | ☒ | For null hypothesis testing, the test statistic (e.g. *F*, *t*, *r*) with confidence intervals, effect sizes, degrees of freedom and *P* value noted<br>*Give P values as exact values whenever suitable.* |
| ☒ | ☐ | For Bayesian analysis, information on the choice of priors and Markov chain Monte Carlo settings |
| ☒ | ☐ | For hierarchical and complex designs, identification of the appropriate level for tests and full reporting of outcomes |
| ☒ | ☐ | Estimates of effect sizes (e.g. Cohen's *d*, Pearson's *r*), indicating how they were calculated |

*Our web collection on statistics for biologists contains articles on many of the points above.*

## Software and code

Policy information about availability of computer code

| | |
|---|---|
| Data collection | TCR sequencing data for the TCGA cohorts were kindly made available by Bo Li et al. Treatment naïve melanoma biopsy TCR sequencing data of the pre-treatment validation cohort analyzed here were downloaded from the original publication accessions. |
| Data analysis | Analyses were performed with GraphPad Prism version 7 (GraphPad Software, La Jolla California USA) or R (with relevant packages, v. 3.4.1, The R Foundation for Statistical Computing, Vienna, Austria). Trimmomatic (v0.36) and STAR (v2.5.1) aligner are published or commercial codes and softwares used for some of the analyses. Diversity was calculated using Renyi index (α=1) as per Spreafico et al. |

For manuscripts utilizing custom algorithms or software that are central to the research but not yet described in published literature, software must be made available to editors and reviewers. We strongly encourage code deposition in a community repository (e.g. GitHub). See the Nature Research guidelines for submitting code & software for further information.

## Data

Policy information about availability of data

All manuscripts must include a data availability statement. This statement should provide the following information, where applicable:

- Accession codes, unique identifiers, or web links for publicly available datasets
- A list of figures that have associated raw data
- A description of any restrictions on data availability

TCR sequencing data for the TCGA cohorts were kindly made available by Bo Li et al. Treatment naïve melanoma biopsy TCR sequencing data of the pre-treatment validation cohort analyzed here were downloaded from the original publication accessions. Seq data for the pre-treatment training cohort can be downloaded from EGA (accession code EGAS00001005201). The authors confirm that, for approved reasons, some access restrictions apply to data containing patient medical records (patient name, name initials, patient date of birth).

# Field-specific reporting

Please select the one below that is the best fit for your research. If you are not sure, read the appropriate sections before making your selection.

- [x] Life sciences
- [ ] Behavioural & social sciences
- [ ] Ecological, evolutionary & environmental sciences

For a reference copy of the document with all sections, see nature.com/documents/nr-reporting-summary-flat.pdf

# Life sciences study design

All studies must disclose on these points even when the disclosure is negative.

| | |
|---|---|
| Sample size | We used the “rule of the thumb” (one-in-10 rule) to determine the maximum number of covariates to use in the regression models to minimise the risk of overfitting (Harrell, F. E. Jr.; Lee, K. L.; Califf, R. M.; Pryor, D. B.; Rosati, R. A. "Regression modelling strategies for improved prognostic prediction". Stat Med. 1984). |
| Data exclusions | Patients with less than 4 TCR sequences identified from RNA-Seq data were excluded from downstream analysis because 4 is the minimum requirement to compute the diversity index. This is explained in the Methods "Samples with less than 4 TCR sequences were outbound for the algorithm to calculate Renyi index and were excluded from the analysis." |
| Replication | Experiments could not be replicated because they were performed with patient-derived samples and the material was not sufficient to repeat the experiments. |
| Randomization | Randomization was not relevant for this study because it's a longitudinal, not interventional biomarker study with a single observational arm. |
| Blinding | The Investigators were blinded to the patient clinical response group during experiments. Clinical response was assessed independently after the experiments and the clinicians were blinded to the experiment results. |

# Reporting for specific materials, systems and methods

We require information from authors about some types of materials, experimental systems and methods used in many studies. Here, indicate whether each material, system or method listed is relevant to your study. If you are not sure if a list item applies to your research, read the appropriate section before selecting a response.

## Materials & experimental systems

| n/a | Involved in the study | |
|---|---|---|
| x | | Antibodies |
| x | | Eukaryotic cell lines |
| x | | Palaeontology and archaeology |
| x | | Animals and other organisms |
| | x | Human research participants |
| | x | Clinical data |
| x | | Dual use research of concern |

## Methods

| n/a | Involved in the study | |
|---|---|---|
| x | | ChIP-seq |
| x | | Flow cytometry |
| x | | MRI-based neuroimaging |

## Human research participants

Policy information about studies involving human research participants

| | |
|---|---|
| Population characteristics | Biopsy pre-treatment samples from Manchester patients were collected under the Manchester Cancer Research Centre (MCRC) Biobank ethics application #18/NW/0092, the study was approved by MCRC Biobank Access Committee application 13_RIMA_01. Biopsy pre-treatment samples and clinical information from Padova patients were collected under the University of Padova Department of Surgery, Oncology and Gastroenterology ethics application 04/03/2002 protocol #448 and Veneto Oncology Institute ethics approval 09/04/2018 protocol #006264. Biopsy pre-treatment samples from Meldola patients were collected under ethics application #5483/2018 of Comitato Etico della Romagna. All patients gave written informed consent to the use of the samples for research purposes. All clinical investigations have been conducted according to the principles expressed in the Declaration of Helsinki and good clinical practice guidelines. A total of 17 patients with metastatic melanoma, treated with either pembrolizumab or nivolumab as first-line therapy as per standard of care were included in the study, and 16 were retained for the analyses; of them, 11 had sufficient material to perform IHC analysis for PD-L1 and single nucleotide variation analysis. Inclusion criteria were the diagnosis of metastatic melanoma, no previous systemic oncological treatment in the neoadjuvant, adjuvant or metastatic setting for melanoma or other cancers, no concomitant therapy with immunosuppressant drugs at enrolment and no synchronous other active malignancies. The clinical characteristics of the patients are reported in Table 1. |
| Recruitment | Patient recruitment occurred during outpatient clinical practice visits, every potential candidate was screened according to the inclusion and exclusion criteria described above in "Population characteristics". We note an enrichment in nodal biopsy as a biopsy site for our pre-PD1 melanoma patient cohort, reflecting the accessibility of this site for surgery; however, we performed a comparison of the T cell and T cell receptor metrics between nodal and other site biopsy origin and did not evidence significant differences so we are reasonably confident that this did not influence our downstream observations. |
| Ethics oversight | Manchester Cancer Research Centre (MCRC), Biobank ethics application #18/NW/0092; University of Padova Department of Surgery, Oncology and Gastroenterology ethics application 04/03/2002 protocol #448 and Veneto Oncology Institute ethics approval 09/04/2018 protocol #006264; Meldola patients were collected under ethics application #5483/2018 of of Comitato Etico della Romagna. |

Note that full information on the approval of the study protocol must also be provided in the manuscript.

## Clinical data

Policy information about clinical studies

All manuscripts should comply with the ICMJEguidelines for publication of clinical research and a completedCONSORT checklist must be included with all submissions.

| | |
|---|---|
| Clinical trial registration | The study does not involve clinical trials/clinical trial associated data, patient samples were collected prior standard of care treatment |
| Study protocol | The study is not a clinical trial but a tumor marker prognostic study |
| Data collection | The study does not involve clinical trials/clinical trial associated data, patient samples were collected prior standard of care treatment |
| Outcomes | Death date was obtained from clinical records or the original publications for the validation cohort. Radiological response was defined as per RECIST criteria and the information was obtained from the original publications. |
